# Three-Dimensionally Ordered Macro/Mesoporous Nb_2_O_5_/Nb_4_N_5_ Heterostructure as Sulfur Host for High-Performance Lithium/Sulfur Batteries

**DOI:** 10.3390/nano11061531

**Published:** 2021-06-10

**Authors:** Haoxian Chen, Jiayi Wang, Yan Zhao, Qindan Zeng, Guofu Zhou, Mingliang Jin

**Affiliations:** 1 National Center for International Research on Green Optoelectronics, South China Academy of Advanced Optoelectronics, South China Normal University, Guangzhou 510006, China; haoxian.chen@ecs-scnu.org (H.C.); qin-dan.zeng@ecs-scnu.org (Q.Z.); guofu.zhou@m.scnu.edu.cn (G.Z.); 2 School of Information and Optoelectronic Science and Engineering, South China Normal University, Guangzhou 510006, China; jiayi.wang@zq-scnu.org; 3 School of Materials Science and Engineering, Hebei University of Technology, Tianjin 300130, China

**Keywords:** Nb_2_O_5_, Nb_4_N_5_, heterostructure, lithium-sulfur batteries

## Abstract

The severe shuttle effect of soluble polysulfides hinders the development of lithium–sulfur batteries. Herein, we develop a three-dimensionally ordered macro/mesoporous (3DOM) Nb_2_O_5_/Nb_4_N_5_ heterostructure, which combines the strong adsorption of Nb_2_O_5_ and remarkable catalysis effect of Nb_4_N_5_ by the promotion “adsorption-transformation” mechanism in sulfur reaction. Furthermore, the high electrocatalytic activity of Nb_4_N_5_ facilitates ion/mass transfer during the charge/discharge process. As a result, cells with the S-Nb_2_O_5_/Nb_4_N_5_ electrode delivered outstanding cycling stability and higher discharge capacity than its counterparts. Our work demonstrates a new routine for the multifunctional sulfur host design, which offers great potential for commercial high-performance lithium–sulfur batteries.

## 1. Introduction

Electronic devices play a vital role in modern society, setting high standards for corresponding energy storage systems [1,2]. Lithium–sulfur batteries (LSBs) are demonstrated as one of the most promising candidates owing to their high theoretical energy density, low cost, and environmental friendliness [3,4,5]. However, the solid-electrolyte interphase (SEI) has been found to have poor mechanical strength and Li-ion conductivity. The formation of unstable SEI causes safety issues and faster decay of capacity in the anode side for LSB [6]. Artificial SEI fabricated by fluorinated electrolyte and ultrathin bilayer SEI are thus applied to protect the electrodes and suppress Li dendrite growth [7,8]. Besides, the development of LSBs is hindered by low conductivity of sulfur and its discharge product, repeated volume change, and severe lithium polysulfides (LiPS) shuttle effect [9,10,11,12].

To enhance the performance of LSBs, several kinds of sulfur hosts have developed by researchers, including carbon materials, conductive polymer, and metallic chalcogenides, among others [13,14,15,16,17]. A sulfur host can fasten charge transfer in LSB, capture LiPS, and catalyze each step of conversion of this chemical species. Qiao et al. combined iron phosphide (FeP) with reduced graphene oxide (rGO) to construct a sulfiphilic composite [18]. The catalytic properties of FeP and the electron transport properties of rGO are integrated by the synergistic effect, which results in high coulombic efficiency and capacity of the cell loaded with this kind of sulfiphilic host. Recently, polar metal oxides were found to deliver great potential to serve as a sulfur host relying on polar-polar interactions with the LiPS [19,20,21]. As an oxygen-rich material, anions of metal oxides work as active sites to absorb the LiPS. Among the family of oxides, Nb_2_O_5_ shows high LiPS anchor ability due to the strong metal-sulfur bond [22,23]. However, as an insulator with a wide band gap energy, the Nb_2_O_5_ sulfur host is still limited by poor electronic conductivity. While, transition metal nitride is reported to possess high conductivity and catalytic activity, and have been widely used as a sulfur host for LSBs [24,25,26,27]. When different catalysts are applied in LSB, them can catalyze various processes in the conversion of LiPS. Wang et al. found that FeP is able to catalyze the liquid-liquid-solid process, while Fe_3_O_4_ can promote the solid-liquid conversion. When the batteries were assembled with these two catalysts, the cycle stability and capacity retention of the battery was improved simultaneously [28]. Hence, it is a feasible way to combine the Nb_2_O_5_ and transition metal nitride to achieve a high-performance sulfur host.

Herein, we develop a three-dimensionally ordered macro/mesoporous (3DOM) Nb_2_O_5_/Nb_4_N_5_ heterostructure, which combines the strong adsorption of Nb_2_O_5_ and remarkable catalysis effect of Nb_4_N_5_, promoting the “adsorption-transformation” mechanism in the lithium-sulfur battery. Furthermore, the high electrocatalytic activity of Nb_4_N_5_ can provide a fast ion transfer routine during the cycling process and the ordered porous structure not only provides sufficient space for sulfur loading, but also improves the electrolyte infiltration. Therefore, the S-Nb_2_O_5_/Nb_4_N_5_ electrode delivers satisfying cycling stability and remarkable discharge capacity. 

## 2. Materials and Methods

### 2.1. Materials Preparation

The polymethyl methacrylate (PMMA) template was prepared according to our reported methods [29]. In the typical procedure of the synthesis of 3DOM Nb_2_O_5_/Nb_4_N_5_, 20 mL ethanol was mixed with 1.35 g of niobium pentachloride (NbCl_5_, Aladdin, Shanghai, China) under magnetic stirring. When a clear solution was formed, the prepared PMMA template was immersed in the precursor solution for 12 h. Subsequently, the precursor solution was removed from the PMMA template through vacuum filtration. The obtained sample was put into a porcelain boat and dried in air at 60 °C. Subsequent calcination at 600 °C in air for 3 h was employed to remove the PMMA template. The obtained 3DOM Nb_2_O_5_ was heated under NH_3_ to prepare 3DOM Nb_2_O_5_/Nb_4_N_5_. 

### 2.2. Characterization

X-ray diffraction (XRD, D8 Focus Bruker, Karlsruhe, Germany), scanning electron microscopy/ energy dispersive spectroscopy (FE–SEM/EDS, ZEISS Ultra 55, Oberkochen, Germany) and transmission electron microscopy (TEM, JEOL 2100, Tokyo, Japan) were employed to observe the phase and morphology of 3DOM Nb_2_O_5_/Nb_4_N_5_. The element value and bonding state were explored by the X–ray photoelectron spectra (XPS, Thermo Scientific ESCALAB 250Xi, Waltham, MA, USA). N_2_ adsorption–desorption isotherms and pore distribution were tested using V-Sorb 2800P. Thermogravimetric analysis (TGA, PerkinElmer TGA-8000, Waltham, MA, USA) was used to determine the sulfur content of the samples. 

### 2.3. Cell Assembling and Testing

All reagents for assembling and testing are purchased from Aladdin (Shanghai, China) without further purification. At a mass ratio of 75:25 (wt.%), Nb_2_O_5_/Nb_4_N_5_ and sulfur powder were ground together and melting-diffusion routine was conducted to obtain S-Nb_2_O_5_/Nb_4_N_5_. N-hydroxy-2-pyrrolidone (NMP) was used as a solvent, and S-Nb_2_O_5_/Nb_4_N_5_ and conductive carbon black with polyvinylidene fluoride powder (PVDF) were mixed to produce a black slurry, in a mass ratio of 8:1:1. Al foil, serving as current collector, was coated with the as-prepared slurry and dried at 60 °C overnight. CR-2032 coin-type cells were applied to study the prepared electrode. To assemble the cells, cathode was made with a diameter of 12 mm. In this kind of cell, Li foil is an anode while Celgard 2400 works as a separator. 1,3-dioxolane (DOL) and 1,2-dimethoxyethane (DME) (*v*/*v* = 1/1) were mixed with 1 M LiTFSI and 1% LiNO_3_ additive to serve as an electrolyte of the cell. Cyclic voltametric test and electrochemical impedance spectroscopy were carried out on a CHI660E (CH Instruments, Inc., Austin, TX, USA) electrochemical workstation. Charge-discharge surveys were conducted on a Neware battery tester (Shenzhen, China) from 1.7 V to 2.8 V. 

LiPS adsorption test: The polysulfides were produced by adding sulfur and lithium sulfides in DME at a specific molar ratio. All tested samples was mixed with diluted Li_2_S_6_ solution inside the glove box. The photographs of absorption result were collected after stirring for 2 h and aging for one-sixth of a day.

Symmetric cells: 0.5 mg cm^−2^ of active materials were dropped onto the circular disks (12 mm in diameter) of carbon cloths. The amount of 0.2 M Li_2_S_6_ solution used in symmetric cells was 30 μL. CV measurements were obtained on a CHI660E (CH Instruments, Inc., Austin, TX, USA) electrochemistry workstation from −0.8 to 0.8 V in 1 mV s^−1^.

Li_2_S nucleation test: 0.25 M Li_2_S_8_ electrolyte was prepared for the test. Nb_2_O_5_/Nb_4_N_5_ heterostructure materials/carbon cloth and the lithium metal were used as electrodes. During the test, all batteries were discharged at 2.06 V with a steady current of 0.112 mA, and then maintained at 2.05 V until the current was decreased to 10^−5^ A.

Linear sweep voltammetry (LSV) test: To investigate the oxidization behavior of Li_2_S, LSV measurements were performed in methanol with 0.1 M Li_2_S. Typically, to construct a three-electrode system, an Ag/AgCl electrode and platinum wire are used as the reference electrode and counter electrode, respectively. Moreover, the glass carbon electrode covered with prepared materials was used as the working electrode. The prepared materials were dispersed in NMP and added onto a glass carbon electrode to fabricate a working electrode. The tests were conducted by scanning from −0.4 to −0.2 V in 5 mV s^−1^.

## 3. Results and Discussion

**Figure 1:**
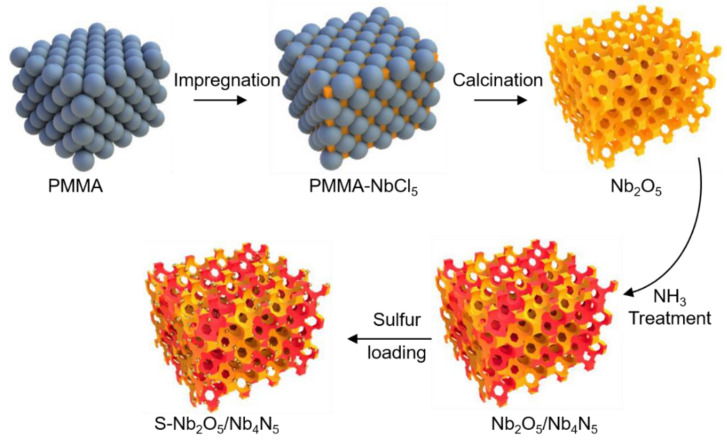
Schematic diagram of preparation of 3DOM S-Nb_2_O_5_/Nb_4_N_5_.

As observed in Figure 1, NbCl_5_ first penetrated the closed-packing PMMA template by capillarity forces. Subsequently, the PMMA was removed by heating at 600 °C, thus by the formation of 3DOM Nb_2_O_5_. The construction of a heterostructure relied on the Nb_2_O_5_ nitridation treatment through NH_3_ erosion at a high temperature. When the conversion of the LiPSs species occurs, satisfactory pores and voids were provided by the unique 3DOM structure, for storing and immobilizing sulfur. Working as a polar material, the Nb_2_O_5_ part in the heterojunction can strongly and chemically adsorb polysulfide. Soon after adsorption, the niobium nitride (Nb_4_N_5_) in the heterojunction enhances the catalysis of polysulfide and solid Li_2_S nucleation is seen.

**Figure 2:**
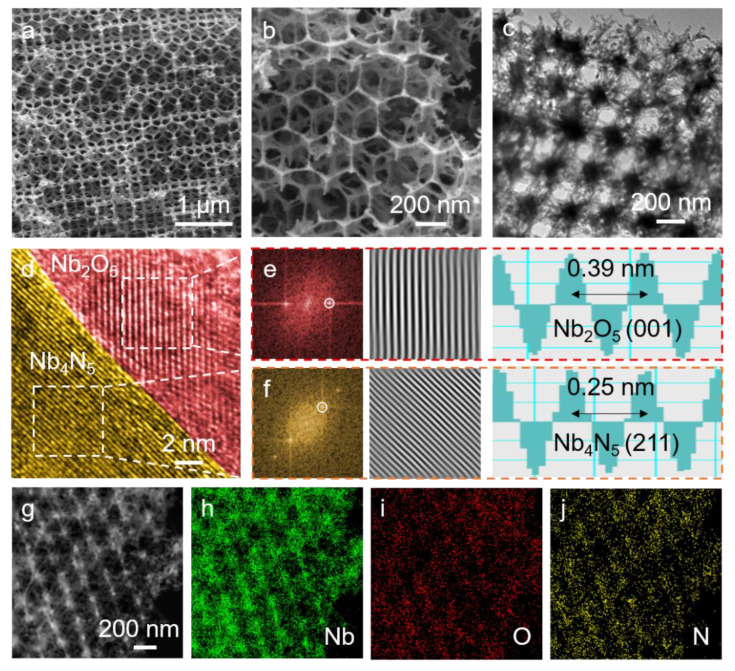
(**a**,**b**) SEM images of 3DOM Nb_2_O_5_/Nb_4_N_5_; (**c**) TEM and (**d**) HRTEM image of 3DOM Nb_2_O_5_/Nb_4_N_5_; (**e**,**f**) FFT patterns, inverse FFT patterns, and lattice spacing images of the selected area; (**g**–**j**) STEM image and the corresponding element distribution of 3DOM Nb_2_O_5_/Nb_4_N_5_.

SEM images of Nb_2_O_5_/Nb_4_N_5_ are shown in Figure 2a,b. Figure 2a shows that the composites have three-dimensionally ordered porous structure and the pores are uniformly distributed and interconnected. The pore diameter is around 150–200 nm (Figure 2b) and sulfur can be absorbed intensely by these nanopores during galvanostatic charge–discharge cycling. Figure S1 shows the energy dispersive spectroscopy (EDS) result. Through the analysis of this mapping, the composites have 24% Nb_2_O_5,_ while the other 76% is Nb_4_N_5_. The TEM result further confirms the 3DOM structure of Nb_2_O_5_/Nb_4_N_5_ in Figure 2c, and implies that the unique structure is preserved during the formation of the Nb_2_O_5_/Nb_4_N_5_ heterostructure. More importantly, the pore diameter is 160 nm, which is consistent with the SEM image. HRTEM image of Nb_2_O_5_/Nb_4_N_5_ (Figure 2d) demonstrates a distinctly different crystalline structure, which is attributed to Nb_2_O_5_ and Nb_4_N_5_. For further verification, fast Fourier transform (FFT) and inverse FFT patterns are collected, as shown in Figure 2e,f. Clear diffraction spots can be seen in both areas, demonstrating the excellent crystallinity of Nb_2_O_5_ and Nb_4_N_5_. The crystal plane spacings are measured to be 0.39 nm and 0.25 nm, which is consistent with typical (001) plane of Nb_2_O_5_ and (211) plane of Nb_4_N_5_. Furthermore, the scanning TEM image of Nb_2_O_5_/Nb_4_N_5_ is shown in Figure 2g–j, confirming uniform element distribution. The above results prove the successful preparation of Nb_2_O_5_/Nb_4_N_5_ heterostructure material.

**Figure 3:**
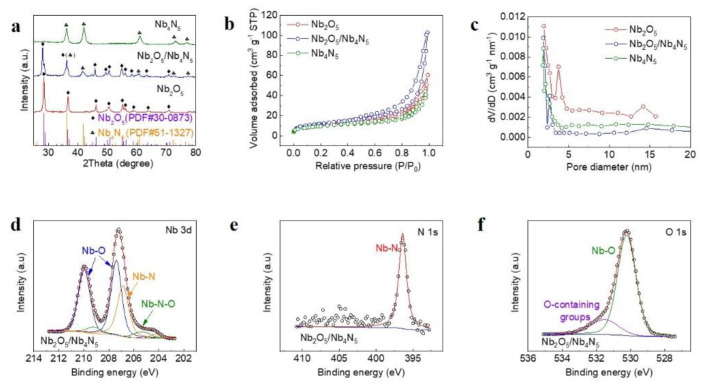
(**a**) XRD patterns; (**b**) N_2_ adsorption/desorption isotherms and (**c**) pore distributions of Nb_4_N_5_, Nb_2_O_5_/Nb_4_N_5_ and Nb_2_O_5_. XPS spectra of Nb_2_O_5_/Nb_4_N_5_: (**d**) Nb 3d, (**e**) N 1s, and (**f**) O 1s.

XRD patterns of Nb_4_N_5_, Nb_2_O_5_/Nb_4_N_5_, and Nb_2_O_5_ are shown in Figure 3a; all the peaks are consistent with Nb_4_N_5_ (PDF#51-1327) and Nb_2_O_5_ (PDF#30-0873), indicating the high purity of the synthesized products. The 3DOM structures are further probed through the N_2_ adsorption/desorption isotherms (Figure 3b); a similar specific surface area is obtained by Nb_4_N_5_ (34.6 m^2^ g^−1^), Nb_2_O_5_/Nb_4_N_5_ (38.9 m^2^ g^−1^), and Nb_2_O_5_ (40.9 m^2^ g^−1^), indicating that the pore structures are well preserved during the phase transformation process. The pore distributions in Figure 3c indicate the existence of abundant micropores and mesopores, which can adsorb LiPS by relying the physical effect. Furthermore, the pore volume in Table S3 indicate that Nb_2_O_5_/Nb_4_N_5_ has a large number of bigger pores, which benefit the storing of sulfur. The XPS test is conducted to explore the chemical bonding environment of Nb_2_O_5_/Nb_4_N_5_. Correction for specimen charging is applied to XPS analysis according to the C 1 s peak at 284 eV. Typical Nb-O (209.9 eV, 207.3 eV) and Nb-N (206.8 eV) bonds are obtained (Figure 3d), indicating the co-existence state of Nb_2_O_5_ and Nb_4_N_5_. The high-resolution N 1s spectrum exhibits one major peak at 396.4 eV, which can be owing to the existence of Nb-N bonds of Nb_4_N_5_ (Figure 3e) [30]. Furthermore, one other major peak appears at 530.6 eV, which can be ascribed to Nb-O bonds of Nb_2_O_5_, and a sub peak of O-containing surface group emerged at 531.5 eV (Figure 3f) [22]. These results further confirm the successful construction of the Nb_2_O_5_/Nb_4_N_5_ heterostructure, which is expected to possess high adsorption and catalysis ability for LiPS.

**Figure 4:**
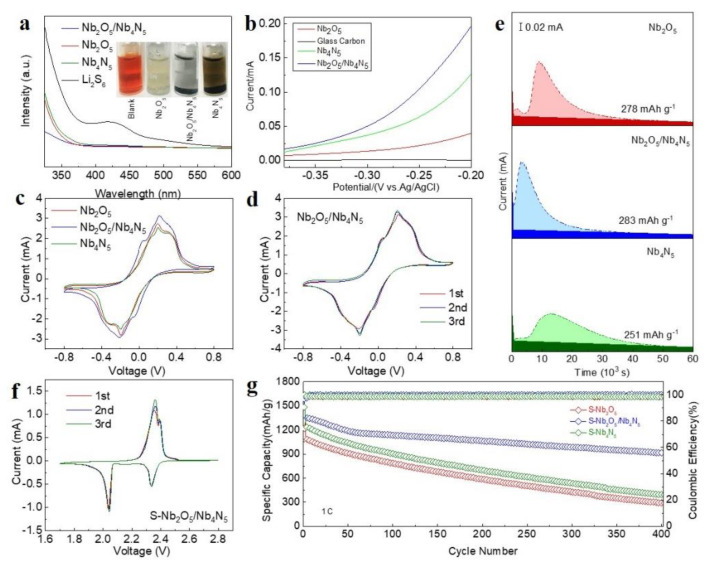
(**a**) LiPS adsorption test; (**b**) LSV test; (**c**,**d**) CV curves of symmetric cell; (**e**) Li_2_S deposition test; (**f**) CV results of the cell with S-Nb_2_O_5_/Nb_4_N_5_ electrode; (**g**) long-term cycling tests at 1 C.

The LiPS adsorption effect of Nb_2_O_5_/Nb_4_N_5_ is evaluated by the LiPS adsorption test using Li_2_S_6_ as a representative LiPS (Figure 4a). As is shown in this photograph, the glass bottles starting from left to right contained blank Li_2_S_6_ solution, 3DOM Nb_2_O_5_ with Li_2_S_6_ solution, 3DOM Nb_2_O_5_/Nb_4_N_5_ with Li_2_S_6_ solution, and 3DOM Nb_4_N_5_ with Li_2_S_6_ solution, respectively. The orange colour in the solution changed to a lighter brown after 3DOM Nb_4_N_5_ was added. Moreover, after 3DOM Nb_2_O_5_ is added into the solution, the color of the solution fades intensely and become much more transparent than that of the one with 3DOM Nb_4_N_5_, verifying that Li_2_S_6_ adsorption ability of Nb_2_O_5_ is much stronger than that of Nb_4_N_5_. At the same time, the solution with Nb_2_O_5_/Nb_4_N_5_ became completely colorless, suggesting the synergistic effect of three-dimensionally ordered porous Nb_2_O_5_ and the unique catalytic nature of Nb_4_N_5_ towards effective trapping of lithium polysulfides. The UV-vis curves comparison displays the vanishing of typical peaks related to S_6_^2−^ and S_4_^2−^, demonstrating the strong adsorption of Nb_2_O_5_/Nb_4_N_5_. The highest current response delivered by the Nb_2_O_5_/Nb_4_N_5_ electrode can be observed in the LSV test (Figure 4b), indicating the enhanced Li_2_S oxidation kinetics achieved by Nb_2_O_5_/Nb_4_N_5_. This result also implies reduction of the energy barrier of conversion of polysulfides by heterojunction, ensuring that the 3DOM Nb_2_O_5_/Nb_4_N_5_ electrodes promoted catalytic process of sulfur [31]. The TGA graph was performed as shown in Figure S2. The S content can reach 73% owing to the abundant hierarchical pore structure. To assess the enhanced electrochemical kinetics in depth, cyclic voltammetry (CV) characterization of the symmetric cells containing the 0.2 M Li_2_S_6_ electrolyte are performed with the scan rate of 1 mV s^−1^ (Figure 4c). The CV profile of 3DOM Nb_2_O_5_/Nb_4_N_5_ exhibits excellent reversibility, with two pairs of redox peaks (−0.03, 0.03 and −0.22, 0.22 V) probed. However, the CV of 3DOM Nb_2_O_5_ and Nb_4_N_5_ only obtains one pair of broadened redox reaction peaks, which is at −0.19 V and 0.19 V, separately. Moreover, the peak intensity of Nb_2_O_5_/Nb_4_N_5_ is higher than that of Nb_2_O_5_ and Nb_4_N_5_, indicating the limited transformation of polysulfides on the bare surface of Nb_2_O_5_/Nb_4_N_5_ heterojunction. The initial three CV cycles (Figure 4d) of the Nb_2_O_5_/Nb_4_N_5_heterojunction are perfectly overlapped, suggesting excellent cycling stability. As is widely accepted by the scientific community, the transformation from Li_2_S_4_ to Li_2_S contributes almost 75% of discharge capacity. As a result, the Li_2_S deposition test is conducted, as shown in Figure 4e. It can be observed from Figure 4e that nucleation peak response of the heterostructure is earlier than that of Nb_2_O_5_ and Nb_4_N_5_, and nucleation capacity of 3DOM Nb_2_O_5_/Nb_4_N_5_ (283 mAh g^−1^) is highest among the three samples. This may lead to a lower overpotential of the nucleation, electrocatalytic conversion of Li_2_S, and adsorbent to polysulfide species [32].

The electrochemical performance is tested by employing the S-Nb_2_O_5_/Nb_4_N_5_ electrode. The CV results are shown in Figure 4f—two distinct reduction peaks and one main oxide peak can be seen. The reduction peaks located at ~2.35 V and ~2.05 V represent the transformation from sulfur to Li_2_S_4_ and further into Li_2_S. The oxide peak is produced by the regeneration of sulfur. The nearly overlapped curves indicate the excellent reversibility of the electrochemical reactions. Figure S3 show the Nyquist plots of the battery loaded with different samples in the frequency range 0.01–100 KHz. The S-Nb_2_O_5_/Nb_4_N_5_ cathode shows the smallest charge-transfer resistance (R_ct_), denoting its fast kinetic process. The internal resistance (R_s_) of all samples is similar and the R_ct_ of S-Nb_2_O_5_/Nb_4_N_5_ cathode is 46.79 Ω (Table S2).Furthermore, a long-term cycling test at 1 C was conducted (Figure 4g). The first 2 cycles at 0.2 C are applied for activation of the electrodes. A high discharge capacity of 1354 mAh g^−1^ at 1 C is obtained and a remarkable reversible capacity of 913 mAh g^−1^ can still be maintained after 400 cycles with a low capacity attenuation rate (0.08% per cycle), which is obviously improved compared with the S-Nb_2_O_5_ and S-Nb_4_N_5_ electrodes. Additionally, the voltage profiles at 1 C are provided in Figure S4; it can be seen that the S-Nb_2_O_5_/Nb_4_N_5_ electrode displays stable voltage plateau and negligible polarization behavior under prolonged cycling. Moreover, cycling test at 1 C with different mass loading is shown in Figure S5. The as-developed S-Nb_2_O_5_/Nb_4_N_5_ electrodes are capable of withstanding at 1 C at sulfur loading of 2 and 6 mg cm^−2^ (Figure S5), attributing the favorable mass/charge transfer and the catalyzed sulfur redox reactions in the Nb_2_O_5_/Nb_4_N_5_ matrix. On comparison of our work with other current works, it is seen that the Nb_2_O_5_/Nb_4_N_5_ electrode exhibits excellent electrochemical performance among recently published heterojunction materials for LSB (Table S4).

## 4. Conclusions

A 3DOM Nb_2_O_5_/Nb_4_N_5_ heterostructure was constructed through in-situ nitridation to serve as a multi-functional sulfur host. The porous structure with interconnected channels can accommodate sulfur as well as facilitate electrolyte infiltration. Strong LiPS immobilization of Nb_2_O_5_ and the remarkable catalysis effect of Nb_4_N_5_ are combined to realize the accelerated LiPS “adsorption-transformation” process. As a result, the LSBs with S-Nb_2_O_5_/Nb_4_N_5_ delivered enhanced kinetics and improved cycling stability and discharge capacity, indicating great capability of Nb_2_O_5_/Nb_4_N_5_ for high-performance LSBs.

## Data Availability

Data is contained within this article and Supplementary material.

## Supplementary Materials

The following are available online at https://www.mdpi.com/article/10.3390/nano11061531/s1, Figure S1: EDS mapping of Nb2O5/Nb4N5, Figure S2: TGA profile of Nb2O5/Nb4N5, Figure S3: Nyquist plots of S-Nb4N5, S-Nb2O5/Nb4N5 and S-Nb2O5, Figure S4: Voltage profiles of S-Nb2O5/Nb4N5 at 1 C, Figure S5: Cycling tests at 1 C with different mass loading, Table S1: Conductivities of Nb4N5, Nb2O5/Nb4N5 and Nb2O5, Table S2: The resistance of Rs and Rct simulated from equivalent circuits, Table S3: Pore size distribution and/or pore volume of Nb4N5, Nb2O5/Nb4N5 and Nb2O5, Table S4: Comparation of electrochemical properties of our work with other works.
