# Supplementary material for: Three-Dimensionally Ordered Macro/Mesoporous Nb2O5/Nb4N5 Heterostructure as Sulfur Host for High-Performance Lithium/Sulfur Batteries
Source: Nanomaterials (Basel). 2021 Jun 10;11(6):1531. doi: 10.3390/nano11061531 (PMC8229476; doi:10.3390/nano11061531)
Supplement: Supplementary file 1 [file nanomaterials-11-01531-s001.zip › nanomaterials-1199665-supplementary.pdf]

Supplementary Materials

Three-Dimensionally Ordered Macro/Mesoporous Nb_2O_5/Nb_4N_5 Heterostructure as Sulfur Host for High-Performance Lithium/Sulfur Batteries

Haoxian Chen [1,†], Jiayi Wang [2,†], Yan Zhao [3,*], Qindan Zeng [1], Guofu Zhou [1] and Mingliang Jin [1,*]

[1] National Center for International Research on Green Optoelectronics, South China Academy of Advanced Optoelectronics, South China Normal University, Guangzhou 510006, China; haoxian.chen@ecs-scnu.org (H.C.); qin-dan.zeng@ecs-scnu.org (Q.Z.); guofu.zhou@m.scnu.edu.cn (G.Z.)

[2] School of Information and Optoelectronic Science and Engineering, South China Normal University, Guangzhou 510006, China; jiayi.wang@zq-scnu.org

[3] School of Materials Science and Engineering, Hebei University of Technology, Tianjin 300130, China

* Correspondence: yanzhao1984@hebut.edu.cn (Y.Z.); mingliang.jin@zq-scnu.org (M.J.)

† H. Chen and J. Wang contributed equally to this work.

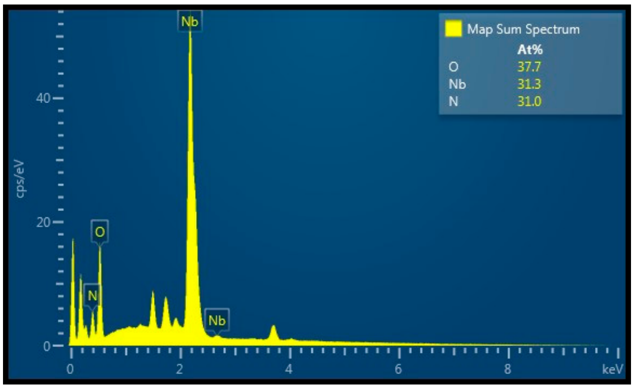

Figure S1. EDS mapping of Nb_2O_5/Nb_4N_5

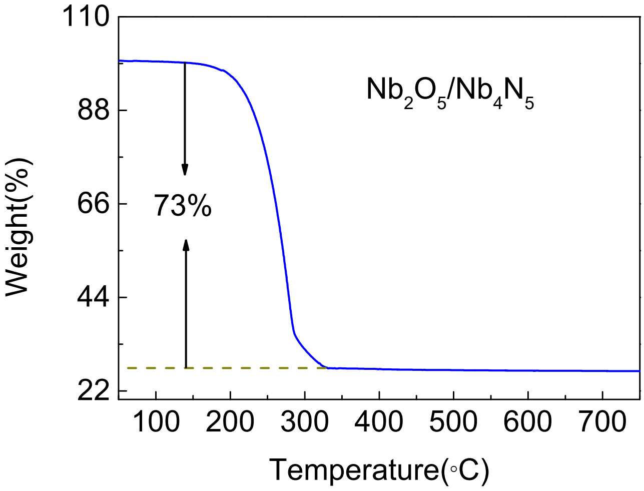

Figure S2. TGA profile of Nb_2O_5/Nb_4N_5

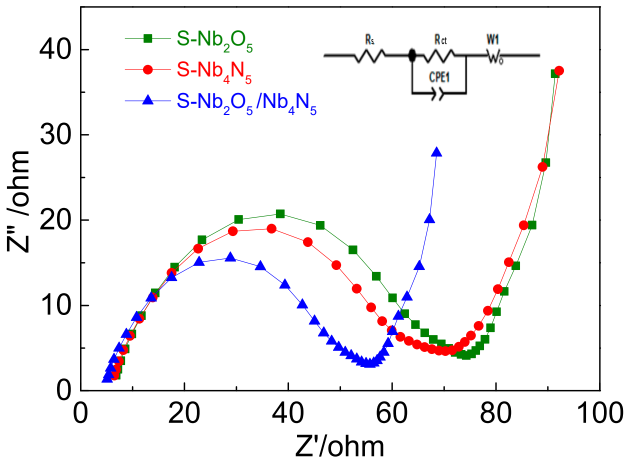

Figure S3. Nyquist plots of S-Nb_4N_5, S-Nb_2O_5/Nb_4N_5 and S-Nb_2O_5

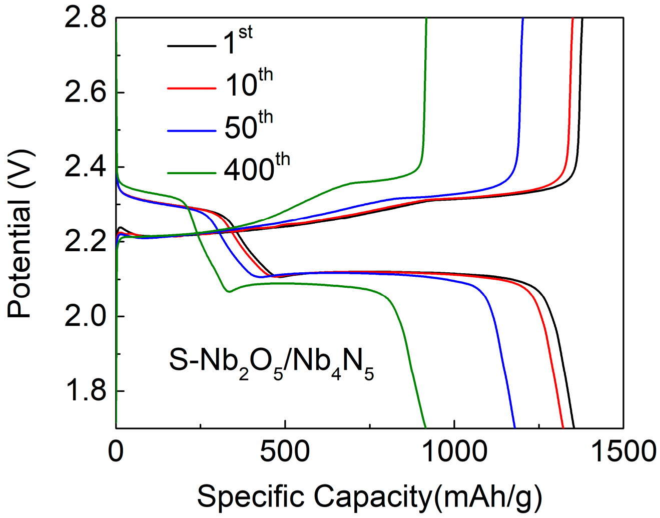

Figure S4. Voltage profiles of S-Nb_2O_5/Nb_4N_5 at 1 C

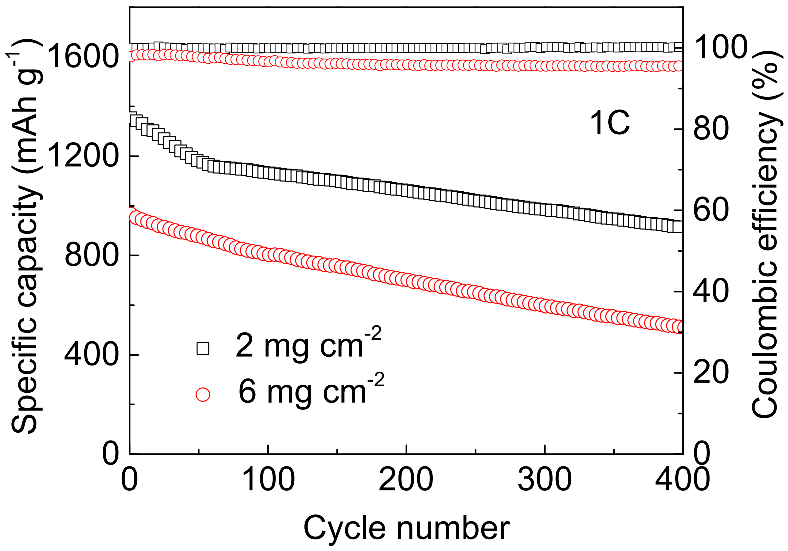

Figure S5. Cycling tests at 1 C with different mass loading

Table S1. Conductivities of Nb_4N_5, Nb_2O_5/Nb_4N_5 and Nb_2O_5

Sample	Conductivity
Nb_4N_5	6.6×10^{-3} S cm^{-1}
Nb_2O_5/Nb_4N_5	4.35×10^{-4} S cm^{-1}
Nb_2O_5	6.91×10^{-6} S cm^{-1}

Table S2. The resistance of Rs and Rct simulated from equivalent circuits

Sample	Rs	Rct
Nb_2O_5/Nb_4N_5	4.48 Ω	46.79 Ω
Nb_4N_5	5.64 Ω	57.55 Ω
Nb_2O_5	5.97 Ω	62.38 Ω

Table S3. Pore size distribution and/or pore volume of Nb_4N_5, Nb_2O_5/Nb_4N_5 and Nb_2O_5

Sample	Pore size distribution	Pore volume
Nb_2O_5/Nb_4N_5	2.7 nm	0.16 cm^3/g
Nb_4N_5	2.1 nm	0.08 cm^3/g
Nb_2O_5	3.8, 14.2 nm	0.05 cm^3/g

Table S4. Comparation of electrochemical properties of our work with other works

Materials	Rate / Specific capacity/Cycle (C)/ (mAh g^{-1})	Reference
Ag/VN@Co/NCNT	2/693/2000	ACS Nano 2021, 15, 4, 6849-6860
Co_9S_8@MoS_2 /CNF	1/803/400	ACS Nano 2020, 14,12,17285-17294
V_8C_7-VO_2	4/410/800	Adv. Mater. 2020, 32, 50, 2005967
TiO_2-Ni_3S_2	0.5/638/900	Adv. Mater. 2020, 32, 32, 2000315
TiO_2-TiN	1/2000/704	Energy Environ. Sci., 2017,10, 1694-1703
Co_9S_8/CoO	1/601/1000	Nano Energy 2019, 60, 332-339
$MoSe_2$/MoO_2	0.5/848/500	Chem. Eng. J. 2020,381, 122672
Nb_2O_5/Nb_4N_5	1/913/400	Our work
